# Young people's perceptions of acne and acne treatments: secondary analysis of qualitative interview data†

**DOI:** 10.1111/bjd.18684

**Published:** 2019-12-25

**Authors:** A. Ip, I. Muller, A.W.A. Geraghty, A. McNiven, P. Little, M. Santer

**Affiliations:** ^1^ Primary Care and Population Science Faculty of Medicine University of Southampton Southampton U.K.; ^2^ Nuffield Department of Primary Care Health Sciences Radcliffe Observatory Quarter Woodstock Road Oxford OX2 6GG U.K.

## Abstract

**Background:**

Acne vulgaris is a common skin condition affecting approximately 95% of adolescents to some extent. First‐line treatments are topical preparations but nonadherence is common. A substantial proportion of patients take long courses of oral antibiotics, associated with antibiotic resistance.

**Objectives:**

This study aimed to explore young people's views and experiences of acne and its treatments.

**Methods:**

We report a secondary thematic analysis of interview data collected by researchers in the Health Experiences Research Group (HERG), University of Oxford. A total of 25 transcripts from young people aged 13–24 years with acne were included.

**Results:**

Acne is often perceived as a short‐term self‐limiting condition of adolescence and this appears to have implications for seeking treatment or advice. Participants widely perceived topical treatments as being ineffective, which seemed related to unrealistic expectations around speed of onset of action. Many participants felt they had tried all available topical treatments, although were unsure what was in them or unaware of differences between cosmetic and pharmaceutical treatments. They had concerns around how to use topicals ‘properly’ and how to avoid side‐effects. They were also concerned about the side‐effects or necessity of oral treatments, although few seemed aware of antibiotic resistance.

**Conclusions:**

People with acne need support to manage their condition effectively, particularly a better understanding of different topicals, how to use them and how to avoid side‐effects. Unrealistic expectations about the onset of action of treatments appears to be a common cause of frustration and nonadherence. Directing people towards accessible evidence‐based information is crucial.

**What's already known about this topic?**

- There is a common perception that acne is a short‐term condition that will resolve without treatment.
- Previous research has shown that nonadherence to topical treatments is common and that oral antibiotics are the most commonly prescribed treatment for acne in the U.K.
- Further research is needed to understand how young people perceive acne treatments and the implications of this for treatment adherence and self‐management.

**What does this study add?**

- People often said they had tried all available topical preparations for acne, but seemed confused between cosmetic and pharmaceutical treatments.
- People seemed unsure how to use topical treatments ‘properly’ or how to avoid side‐effects. This was rarely discussed with health professionals.
- People's perception of acne as a short‐term condition appeared to influence their expectations around onset of action of treatment and their views about its effectiveness and necessity.

**What are the clinical implications of the work?**

- The perception of acne as a short‐term condition has implications for self‐management and motivation to seek and adhere to treatments.
- Providing advice about onset of action of treatments and how to prevent side‐effects is crucial, including directing people towards accessible, written, evidence‐based information.
- People's confusion about the different topical treatments available may be alleviated by such information, or by encouraging photos or other recordings of treatments tried and for how long.

**Linked Comment:** Prior. Br J Dermatol 2020; 183:208–209.

## Abstract 2

**Linked Comment:** Prior. Br J Dermatol 2020; 183:208–209.

Plain language summary available online

Acne vulgaris (herein ‘acne’) is a common skin condition affecting up to 95% of adolescents to some extent.^1^ It has the potential for substantial effects on quality of life (QoL).^2^ Due to its significant impact on QoL and its potential to persist or recur for many years, it can be considered a long‐term condition.^2^ Despite this, there is a common perception among the lay and medical community that acne is a teenage condition that will go away on its own.^3^

First‐line treatments for mild‐to‐moderate acne are topical preparations including benzoyl peroxide, retinoids and antibiotics.^1^ However, nonadherence to these is common for reasons including side‐effects, young age, forgetfulness,^4^ insufficient knowledge about acne and the use of medication, cost^5^ and the need for continuous treatment over several weeks before onset of action.^6^ Many people progress to taking oral antibiotics: a recent study found that oral antibiotics were the most commonly prescribed acne treatment in the U.K., and in most cases, a nonantibiotic topical was not prescribed in combination.^7^ They found that oral antibiotics alone were prescribed the most during the index consultation (24·9%).^7^ Co‐prescribing of nonantibiotic topical treatments alongside oral antibiotics is recommended to avoid antibiotic resistance in *Propionibacterium acnes* (*P. acnes*). This is necessary as antibiotic resistance in *P. acnes* is increasing and has become a public health concern.^8^

To date, most research exploring prescribing patterns in acne^7^, ^9^ and adherence to acne treatments has been quantitative.^10^, ^11^, ^12^ Qualitative research would be helpful in exploring perceptions of treatments and reasons for nonadherence but qualitative research on acne has focused mainly on the following: psychological impact,^13^, ^14^ psychosocial impact,^15^, ^16^ experiences of living with acne,^17^, ^18^, ^19^, ^20^, ^21^ causes of acne,^22^ sexual life and acne,^23^ ambivalence and ambiguity in young people's experience of acne,^24^ complementary and alternative medicines for acne^25^ and patients’ relationships with their doctors.^26^ There have been some qualitative studies exploring barriers to adherence to acne treatment,^27^ people's views of topical treatments,^21^, ^28^ oral antibiotics^29^ and perceptions of oral isotretinoin.^30^ However, many of these studies were carried out in other countries,^21^, ^27^, ^30^ and so may not be applicable to our population, and others were exploring barriers to nonadherence only briefly, as their primary focus was on QoL^28^ or the psychological impact of treatment.^30^ This qualitative study was carried out to further understand young people's perception of acne and, in particular, their views and concerns about some of the most commonly prescribed acne treatments, including oral antibiotics and topical treatments for acne.

## Methods

### Design and data collection

This paper is based on a secondary analysis of qualitative interview data from young people with acne. Secondary analysis of existing datasets is increasingly seen as an effective way of maximizing knowledge and potential health benefits.^31^ The data were originally collected by researchers in the Health Experiences Research Group (HERG) at the University of Oxford as part of a wider study funded by National Institute for Health Research (NIHR) for the Research for Patient Benefit programme (Grant Reference Number: PB‐PG‐0213‐30006). They aimed to explore the information and support needs of young people with acne, eczema, psoriasis and alopecia. A qualitative researcher (A.M.) carried out in‐depth, semi‐structured interviews with 97 young people aged 13–25 years in England between October 2014 and December 2015. This included 25 interviews with people with acne.^24^ The duration of each interview was approximately 2 h. Participants were recruited via social media platforms including Facebook and Twitter; patient and public platforms; primary care; secondary care; and universities, colleges and schools. The inclusion criteria for the study was people aged 13–25 years with acne in England. Researchers did not screen participants based on their duration of acne or severity although participants usually discussed this in the interviews. A sampling matrix was used to produce a maximum variation sample consisting of a range of demographic factors including age, ethnicity, sex and occupation. This was to ensure that there was a variety of perspectives included in the research. The interviews were recorded using video or audio and transcribed verbatim for analysis.^32^ The University of Oxford, which holds the copyright for the interviews, has given permission for data sharing and this paper is a re‐analysis of the interview data.

Further information about the recruitment and data collection process can be found in the paper by McNiven.^24^ Further analyses and extracts from the interviews can also be seen on the platform healthtalk.org.^32^ Ethical approval for the original research project was obtained by Berkshire NRES Committee South Central.

### Data analysis

All 25 transcripts were uploaded onto NVivo 11 software, which was used to manage the data. An inductive thematic analysis was carried out, drawing on aspects of Joffe and Yardley's^33^ approach (using a coding framework for the team to review) in combination with analytic steps as recommend by Braun and Clarke.^34^ This involved repeatedly reading the transcripts to gain a comprehensive understanding of the data, carry out line‐by‐line coding, generate themes by combining and splitting collated codes, and using a coding framework to give definitions and labels to a set of codes. Three transcripts were coded independently by two members of the research team (A.I. and M.S.), and the coding manual was discussed and developed with the research team consisting of psychologists and general practitioners (GPs). The coding manual was then used to code the remaining transcripts and thus the framework was iteratively revised throughout the analysis. Negative cases were sought and data saturation of main themes was reached as no new information was observed.

## Results

A total of 25 transcripts were analysed. The sample consisted of 18 women and seven men, with an age range of 13–24 years (median and mode average age of 20 years). The sample consisted of different severities and time with condition ranged from a few months to 13 years. Sample demographics are shown in Table 1.

**Table 1:** Sample demographics

Variable	Frequency, *n* (%)
*Sex*	
Male	7 (28)
Female	18 (72)
*Age (years)*	
13–15	3 (12)
16–24	22 (88)
Mean ± SD	20 ± 2·9
*Occupation*	
Postgraduate student	2 (8)
School/college/undergraduate student	21 (84)
Other	2 (8)
*Ethnicity*	
White British	16 (64)
White Greek	2 (8)
White Hungarian	1 (4)
White Dutch	1 (4)
White Other	1 (4)
Chinese	4 (16)

**Figure 1:**
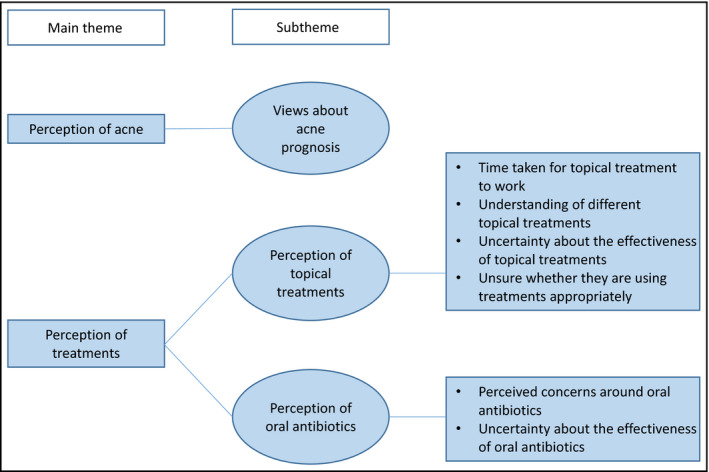
Diagram illustrating the overarching themes discussed in this paper.

Three overarching themes were identified from the analysis. In preparing this paper we focused on two of these themes (Perception of acne and Perception of treatments) and the following subthemes that fall under these overarching themes, as they appeared particularly important in relation to treatment adherence: views about acne prognosis; perceptions of topical treatments; and perceptions of oral antibiotics (see Fig. 1).

### Perception of acne: views about acne prognosis

Participants spoke about puberty as one of the main causes of their acne. This appeared to make young people feel that acne should be a short‐term condition, when in their experience it often followed a more chronic course over years. Some participants said they had initially been less concerned about their acne as they expected to ‘grow out of it’. This expectation often led to frustration and confusion when it was not met.

*‘My skin was just as–, just got bad again and I was, you know, I'd turned 24 and I was just really stressed out about it because I thought [um], you know, I'm 24, this is the sort of thing that typically you associate with teenagers, with adolescence, and I, I guess it just got me down quite a lot. [um] So that wasn't really that fun.’* (Participant 18: female, 24 years)

*‘So I've had acne, I worked out, more than half my life now. Started to flare up probably in Year 8 at school. Really quite badly, to begin with. [Um] But at that point I was thinking “Oh well this is great, I'll get it out the way now, and I'll have beautiful skin by the time I'm, I'm going to sixth form.” And here I am at 25, still [points to jaw line] having it flare up and be part of, part of my daily life. [Um] I have been to various doctors about it.’* (Participant 21: female, 24 years)

For some, their expectations regarding the duration of acne was influenced by other people's experiences. These expectations and the resulting level of concern appeared to have an impact on their treatment decisions and whether treatment was deemed necessary. This had either a positive or a negative impact on seeking treatments, depending on whether acne appeared to have resolved spontaneously for others, or not.

*‘I mean I'd known it from my elder brother having it [um] but he hadn't actually used medication and he'd had it quite severely but it had been like for a short period of time. So I was kind of like “that would be the same for me” like, I based my experience on that and I was like “maybe I'm just gonna have it for a short period of time”.’* (Participant 16: female, 20 years)

*‘I thought, I thought it was like a teenage thing. And everyone says that when you reach, when you reach a certain, a certain age – the acne will go away. But like, looking at my friends who have serious acne [erm] from teenagehood that's just not true. Like you actually do have to take something to like help it go away.’* (Participant 17: female, 20 years)

### Perception of treatments: topical

#### Time taken for topical treatment to work

Participants spoke about feeling frustrated and annoyed with topical treatments as they perceived them as ineffective, possibly because they expected them to work ‘instantly’. This was linked to a process of ‘trial and error’ of going back to their GP for alternative treatments because they wanted something they thought would be more effective. This often resulted in participants trying what they perceived as stronger treatments, such as oral antibiotics.

*‘I decided that I'd rather just go straight back to the stronger [um] medication than have to spend a a long time trying the topical creams which I was pretty sure weren't gonna… I mean maybe they would work but it would just take [er] a lot longer than if I went back to the treatment I was on before that was actually working.’* (Participant 13; male, 16 years)

*‘I can't really remember the names of them I'm afraid. I think both of us expected results more quickly than they were plausibly going to come and so there was quite a pattern of kind of trying something for a couple of weeks and then just moving on to the next thing.’* (Participant 9; female, 22 years)

One participant was told that her topical treatment might take a while to work, which appeared to help her to feel less ‘disheartened’ at the absence of ‘instant’ results.

*‘So I was never too… and that's always been expressed to me which I think is helpful, is that there are very few medications which are like an overnight [um] cures because I think once you do, you know, get the courage up to go to a doctor and then if you're un‐, non‐, if you're not aware that it's not gonna be fixed overnight it's probably quite disheartening when, you know, you go maybe a month and you can't see a change. [um] But I mean you really just have to keep at it because I don't know why but the medication doesn't work instantly. [Um] but that's always been stressed to me which has been really helpful.’* (Participant 16; female, 20 years)

#### Understanding of different topical treatments

Frustration with the trial and error process seemed to be influenced by people's perception that they had tried all the topical treatments available. They described their treatment as ‘the one from the pharmacy’ or ‘the one my mum bought me’. With some assuming that branded topical products such as ‘Clearasil’ were the same as topical treatments prescribed by the GP, possibly leading them to wish to try other medications such as oral antibiotics as a first‐line treatment. Many participants either said they were confused, or appeared to be confused, with regard to the different functions, ingredients and medical names of topical treatments, frequently getting them mixed up with one another.

*‘I'm not sure, I've had loads, I've had like benzoyl something, I think I've had. [er] I'm not sure what this one was called actually, I've pretty much been on everything so you can factor it was like one of them. [laughs] [er] so I'm, I'm not sure what it was actually called.’* (Participant 16; female, 20 years)

#### Uncertainty about the effectiveness of topical treatments

Many participants were uncertain whether topicals were effective, as they reported how they were only ‘keeping their acne at bay’. In addition, some participants spoke about how discontinuing treatment once their skin was clear, or because of side‐effects, would make their acne worse and therefore perceived treatment as ineffective because they had been hoping for a ‘cure’.

*‘I remember the problem was that it tended to make my skin really dry after a while, so you had to stop the treatment. But that was the best one that was working for me. So it really wasn't, so I did it like for a month or two and then my skin got really dry. Then I stopped for a month and then it like came back. So you're just repeating it over and over and over again. You're not really getting a definite cure. So that's, yeah, that's annoying.’* (Participant 8; male, 22 years)

For some, keeping the acne under control was not enough and they were seeking something that would clear it completely.

*‘It was kind of just sort of a keeping it at a certain level as opposed to absolutely like clearing your whole skin and making it sort of a lot better.’* (Participant 4: male, 20 years)

#### Unsure whether they are using topical treatments appropriately

Some participants expressed confusion or misconceptions about how to use topicals properly, including how long to use it for, how much to apply, to which areas and how to manage side‐effects. Only one participant said that they had consulted their GP about uncertainties to resolve the issue and manage it appropriately.

*‘But then I think my doctor did eventually give me one that was a bit more moisturizing. [um] And I also learnt to use like slightly less [laugh]. I think there's always a temptation and most of it is that you use a lot of the product, when actually the recommendation is to use like a pea‐sized amount and I was probably using like a handful. [laughs] So it was probably exacerbated by that.’* (Participant 25: female, 20 years)

Instead, many participants looked at information online and reported stopping topical treatments and progressing to oral treatments. This decision was usually influenced by people's perception that tablets were easier, more effective and quicker acting than topical treatments.

*‘But then over time it just kind of didn't work anymore. And I don't know if I was using it wrong or whatever, but we just decided that it would probably be easier to go for like a tablet, because you can't really do wrong with a tablet.’* (Participant 1: male, 15 years)

### Perception of treatments: oral antibiotics

#### Perceived concerns around oral antibiotics

Some participants spoke about concerns they had about oral antibiotics including feeling ‘queasy’, stomach aches and sun sensitivity. Few participants seemed aware of antibiotic resistance. Despite having equal opportunity to speak about concerns and side‐effects of treatment, only one participant explicitly mentioned it and a few made references to it. These included comments about feeling worried that it might not work for other illnesses in the future and one participant reporting it as a ‘mental or social side‐effect’. The majority of participants seemed less concerned as they reported choosing oral medication when given the choice.

*‘But that also made me feel kind of rubbish because [um] I was becoming more aware of the fact that taking antibiotics you almost feel like you don't really need them when it's your skin [um] and like you kind of want to save it for when you really need it [um] because I mean obviously that's a growing problem and actually like whenever I went to the GP or anyone that had a list of the medication I was on, it's the first thing they would comment on like “Do you really need that like your skin looks alright” but was my skin looking, you know, I mean like alright, alright or a bit worse than it is now [um] you know, was it okay because I was taking medication, I don't know. But [um] and I guess it's their responsibility to do that [um] you know but [um] that also kind of, makes it's feel less satisfactory.’* (Participant 22: female, 20 years)

*‘I'm not sure, to be honest. Because you don't want to make it all over the counter and have everyone taking everything under the sun, especially when it's the antibiotics that tend to work. You don't want to contribute to the already prolific antibiotic resistance that we've got.’* (Participant 21: female, 24 years)

Some participants perceived oral antibiotics as a ‘long‐term treatment’ that should require more supervision from their GP. They also perceived oral treatments as ‘stronger’ and didn't feel that their skin was severe enough to warrant use.

*‘Because like when you're taking tablets – you–, I didn't feel at the time that it was necessary. Because I just thought like “it's j–, it's just on my skin. I can sort it out externally”. And I didn't really want to take medication.’* (Participant 20: female, 17 years)

*‘I didn't necessarily want to be on antibiotics for that long period of time without feeling that like I was, had doctor who was kind of continually aware of it and since, yes since I was initiating the check‐ups that didn't really feel like that, that way to me and I kind of feel a bit more comfortable about creams than [um] pill medication.’* (Participant 16: female, 20 years)

#### Uncertainty about the effectiveness of oral antibiotics

The majority of participants felt that oral antibiotics were not an effective treatment. Participants reported that they were not effective at all or they helped initially but stopped working, which they found frustrating. Participants did not attribute this to potential antibiotic resistance.

*‘I went back to the doctors and they put me on lymecycline again. [um] I think that's around Christmas time and it just didn't work [um] so I was a bit frustrated about that because it had once worked really, really well.’* (Participant 18: female, 24 years)

## Discussion

Young people's perception of acne was that it was a short‐term condition caused by puberty, which they initially expected they would soon grow out of. They felt frustrated when this did not happen and this also had implications for self‐management as they initially did not seek treatment when they thought it a self‐limiting condition. Young people spoke about expecting ‘instant’ results from treatments and perceived them as ineffective when this did not happen. Participants also spoke about not understanding how to use their topical treatments ‘properly’ or how to manage side‐effects, which also led to them stopping treatments. Few reported having consulted with their GP or pharmacist for advice about managing side‐effects or how to use treatments. Young people perceived that they had tried all the topical treatments available as they found it difficult to differentiate between cosmetic, over‐the‐counter and prescribed topical treatments. Few participants mentioned awareness of antibiotic resistance and many opted for oral antibiotics over topical treatments when given the choice.

Previous research has also found that young people believe that acne is commonly viewed as ‘an insignificant feature of adolescence’ and not necessarily a reason to seek medical help.^24^ Through a secondary analysis of the data, this paper explores the implication that people's perception of acne as a short‐term condition may influence their approach to self‐management and treatment adherence. People's perception that acne was a short‐term condition can potentially explain why participants felt frustrated, stopped topical treatment early or opted for alternative treatments in the absence of ‘instant’ results. A recent qualitative study also found that one of the most important attributes of topical treatment was that it would be fast acting, although they do not suggest why participants held this belief.^28^ In this present study, when participants were told that treatment would take time to work this seemed to help them feel less ‘disheartened’. Experts have advised that GPs should spend time explaining that treatment will not work straight away to encourage treatment adherence.^5^, ^35^

We found that young people were often confused about which topical treatments they had tried. A recent cross‐sectional study with university students in Saudi Arabia found that 58·7% of students did not know the name of their prescribed acne medication.^9^ Our findings suggest that, in addition to this, young people find it difficult to differentiate between cosmetic, over‐the‐counter and prescribed acne treatments. This perception often resulted in them trying alternative treatments as first‐line treatment or not using their treatment appropriately. Our findings also showed that many participants stopped topical treatments as they were confused about how to use them appropriately, including managing side‐effects, and only one participant reported consulting a health professional about uncertainties. Fabbrocini *et al*.^28^ similarly found that people reported that the most important attribute for using topical treatments was no side‐effects, particularly bleaching and irritation, but it was not clear from their study why participants were experiencing side‐effects, how they were managing these, and where they were seeking advice. Williams *et al*.^2^ recommend that patients should be told to expect initial irritation and advised about how to minimize this, including applying once every other day initially and using more moisturizing creams. However, we found that instead of consulting a health professional, some participants spoke about using the internet for information, which could potentially exacerbate confusion, as shown in a previous study looking at views about oral antibiotics in online discussion forums.^29^ This suggests that accessible, evidence‐based online information is necessary. Indeed, this secondary analysis is based on data that was collected and used by HERG to produce such a resource, available on www.healthtalk.org.^32^

Many participants in this study reported choosing oral antibiotics as a first‐line treatment when offered a choice. Previous studies have also found that the majority of participants are prescribed oral antibiotics as first‐line treatment but do not discuss the reasons why.^7^, ^36^ Our findings showed that there was a common perception that tablets work faster, are more effective and are easier to take than topical treatments. There is also the possibility that as few participants reported awareness of antibiotic resistance they had fewer concerns over its use and were therefore more likely to use the treatment.

Theoretically, our findings link closely with the Extended Common Sense Model of Illness (ECSM).^37^ The ECSM highlights a range of beliefs about both health conditions and treatments that are related to medication adherence.^37^ These include beliefs about the duration and causes of a condition, along with beliefs about necessity and concerns regarding medication.^37^ In the present study, beliefs regarding the duration of acne (short‐term) and concerns about treatment ineffectiveness (topicals) appeared to prevent engagement with treatments. The ECSM may be a useful model when developing interventions to improve the management of acne and reduce the burden of the condition.

The strength of this study is that it goes into more depth in exploring perceptions of treatments and barriers to treatment adherence than previous qualitative research. The use of maximum variation sampling was useful for identifying common patterns across diverse contexts. However, it can also be seen as a limitation as previous literature suggests that demographic factors including age,^38^ sex^39^ and culture^40^ may influence people's perceptions about their acne. For example, the majority of participants in the study were female, so the inclusion of more males could have influenced the findings. However, the predominance of females is explained by the fact that females are more likely to seek help for their acne than males.^41^ Most participants in this study had more persistent acne so there is a possibility that the inclusion of more people with recent‐onset acne may have reached different findings. As with all secondary analyses of qualitative data, there was no opportunity to further prompt and explore themes identified in early analysis. However, the interviews provided rich data around experiences and views of treatments and we found that data saturation was reached for the main themes of interest.

In conclusion, we found a range of views about topical treatments and oral antibiotics that could lead to early termination of treatment and progression to alternative treatments for acne. It is important for patients to be told that acne can be a chronic condition which requires either long‐term and/or maintenance therapy to reduce the potential for relapse. Our findings highlight the importance of health professionals directing patients towards high‐quality evidence‐based information about acne, particularly to help them understand treatment options, emphasize the delayed onset of action of many acne treatments and how to mitigate against side‐effects. Future research is needed to identify the most effective ways in which support can be provided for young people with acne to help them manage the condition.

## Supporting information


**Powerpoint S1** Journal Club Slide Set.
