# Supplementary material for: Young people's perceptions of acne and acne treatments: secondary analysis of qualitative interview data
Source: Br J Dermatol. 2019 Dec 25;183(2):349–56. doi: 10.1111/bjd.18684 (PMC7496424; doi:10.1111/bjd.18684)
Supplement: Supplementary file 1 — Powerpoint S1 Journal Club Slide Set. [file BJD-183-349-s001.pptx]

## Slide 1
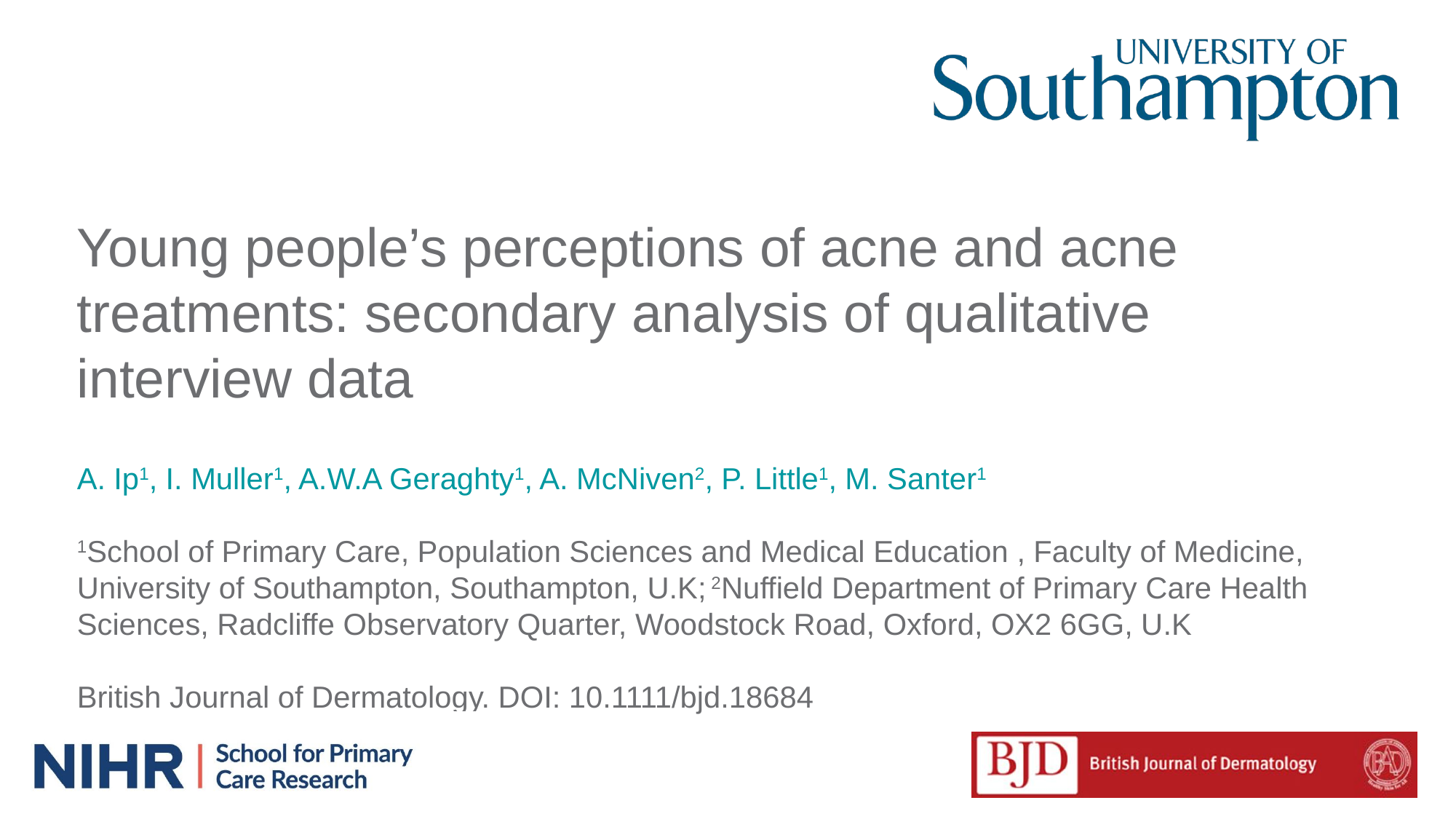

# Young people’s perceptions of acne and acne treatments: secondary analysis of qualitative interview data
A. Ip1, I. Muller1, A.W.A Geraghty1, A. McNiven2, P. Little1, M. Santer1
1School of Primary Care, Population Sciences and Medical Education , Faculty of Medicine, University of Southampton, Southampton, U.K; 2Nuffield Department of Primary Care Health Sciences, Radcliffe Observatory Quarter, Woodstock Road, Oxford, OX2 6GG, U.K
British Journal of Dermatology. DOI: 10.1111/bjd.18684

## Slide 2
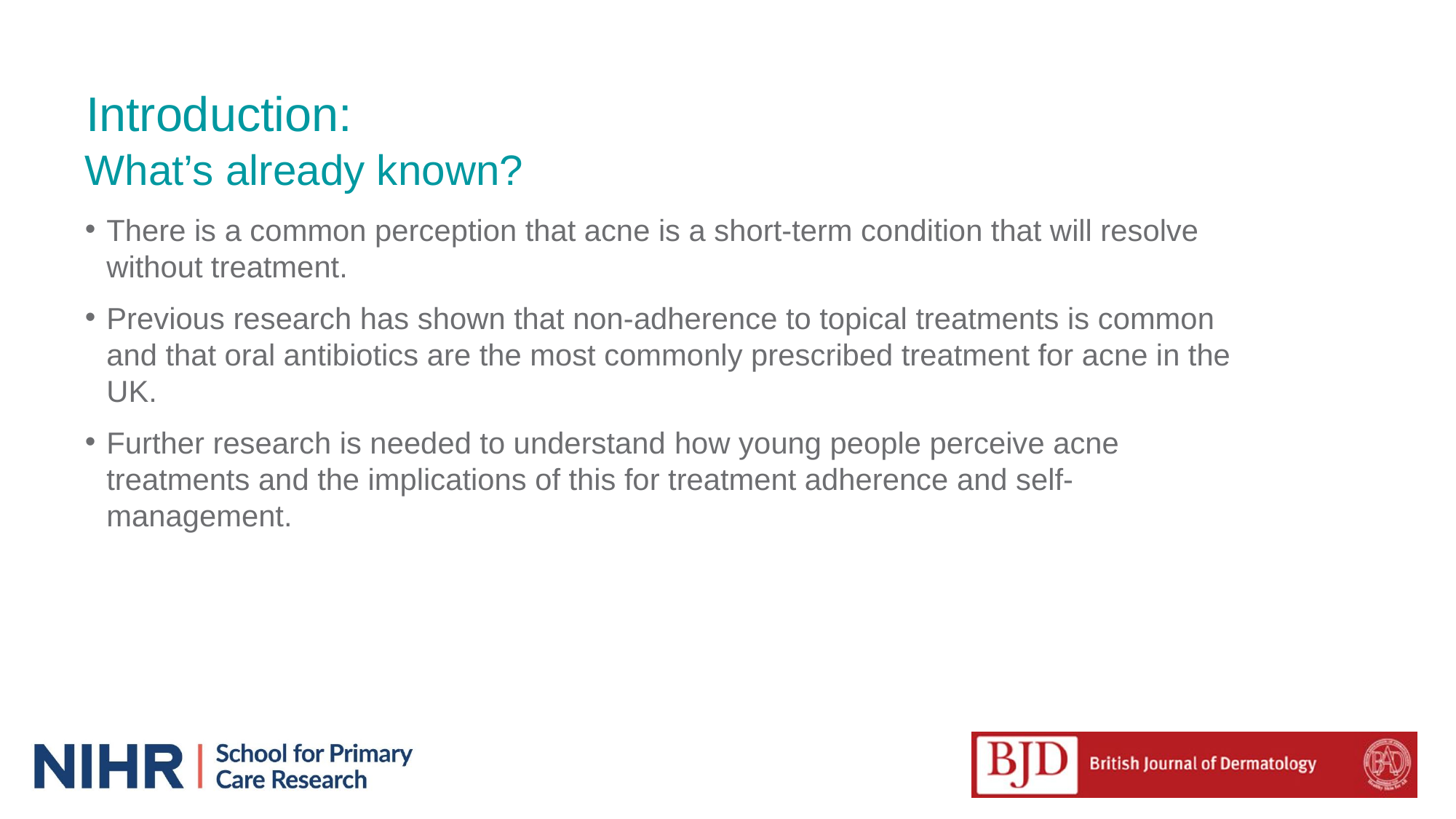

# Introduction:
What’s already known?
There is a common perception that acne is a short-term condition that will resolve without treatment.
Previous research has shown that non-adherence to topical treatments is common and that oral antibiotics are the most commonly prescribed treatment for acne in the UK.
Further research is needed to understand how young people perceive acne treatments and the implications of this for treatment adherence and self-management.

## Slide 3
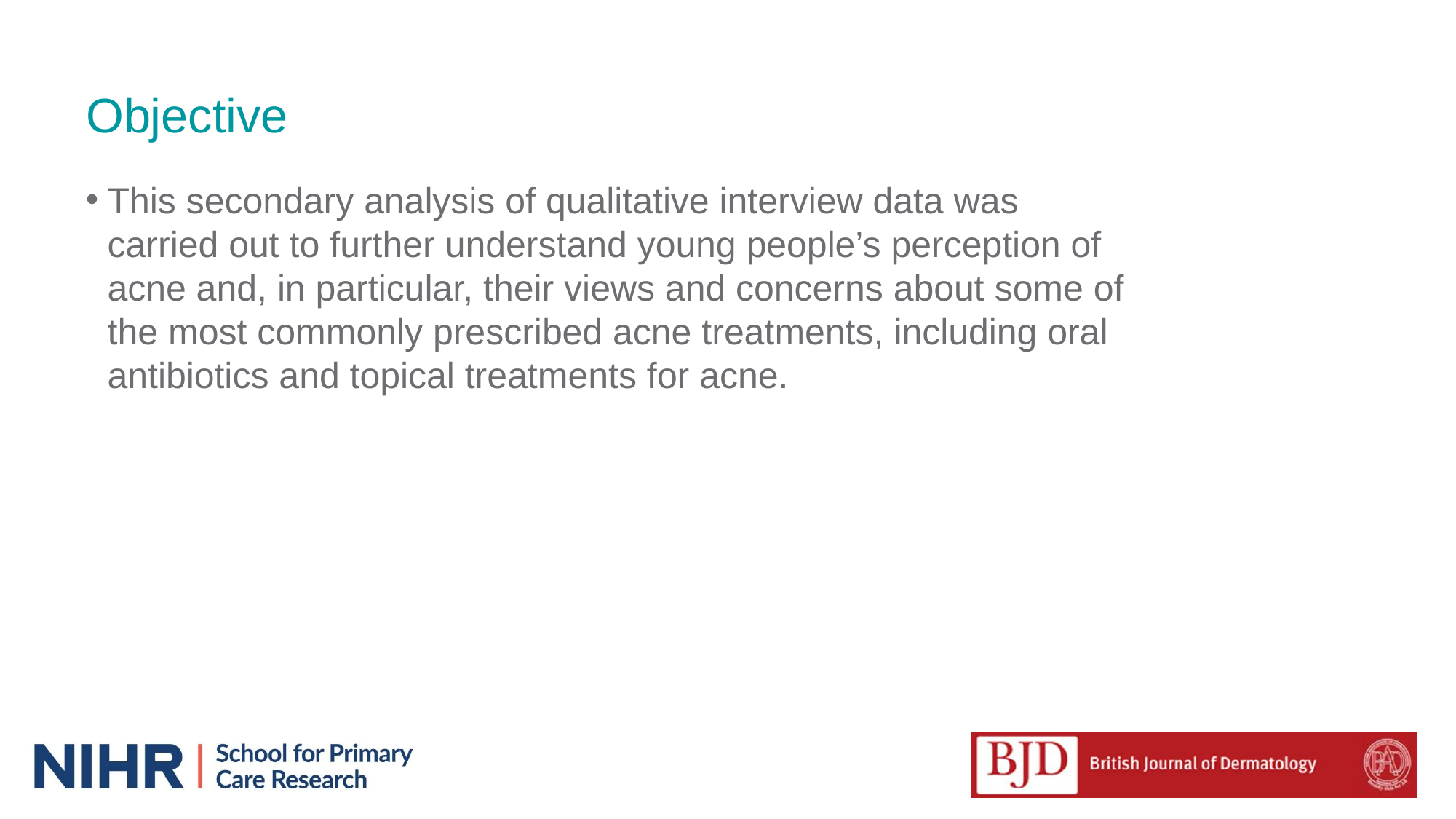

# Objective
This secondary analysis of qualitative interview data was carried out to further understand young people’s perception of acne and, in particular, their views and concerns about some of the most commonly prescribed acne treatments, including oral antibiotics and topical treatments for acne.

## Slide 4
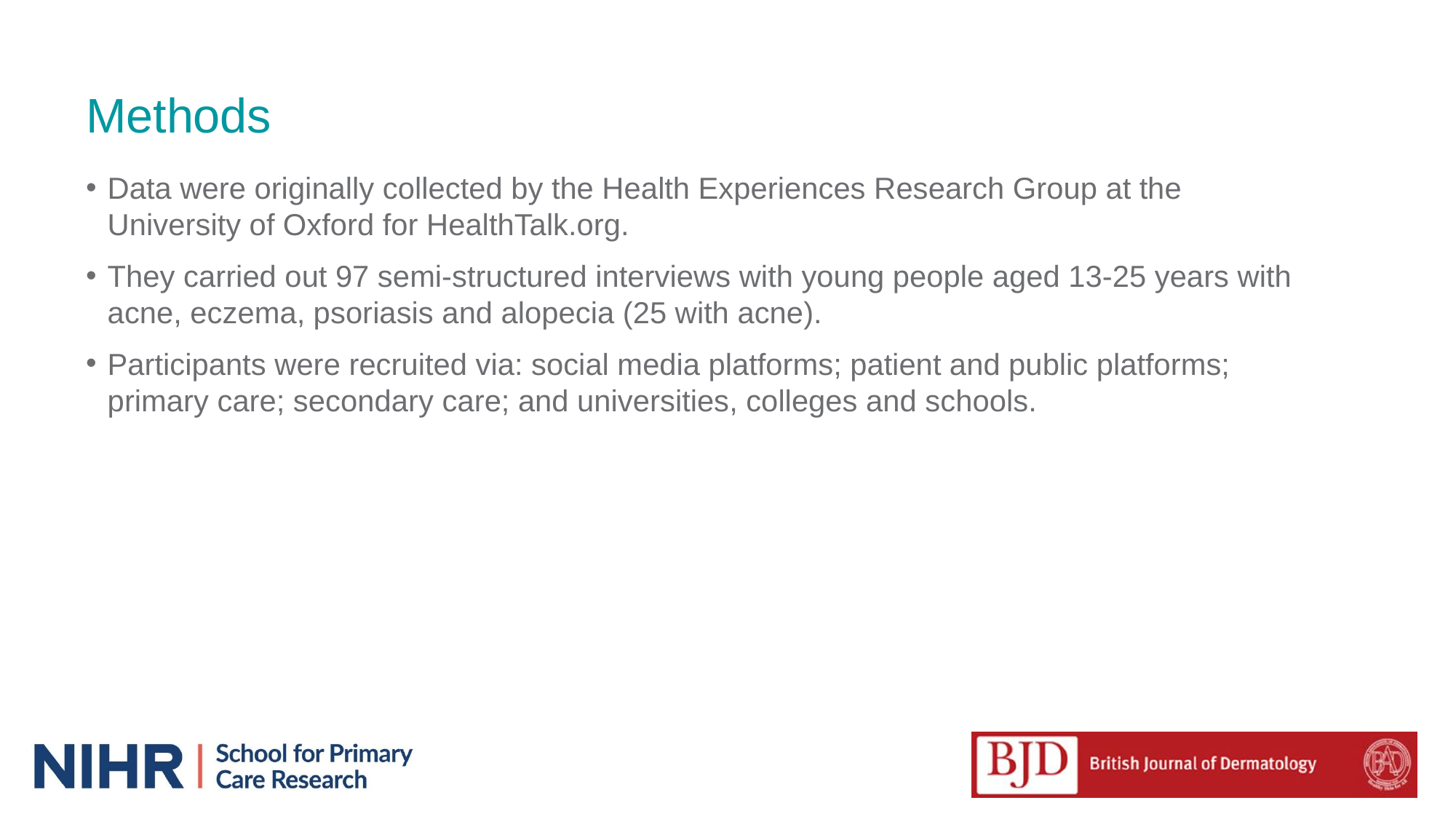

# Methods
Data were originally collected by the Health Experiences Research Group at the University of Oxford for HealthTalk.org.
They carried out 97 semi-structured interviews with young people aged 13-25 years with acne, eczema, psoriasis and alopecia (25 with acne).
Participants were recruited via: social media platforms; patient and public platforms; primary care; secondary care; and universities, colleges and schools.

## Slide 5
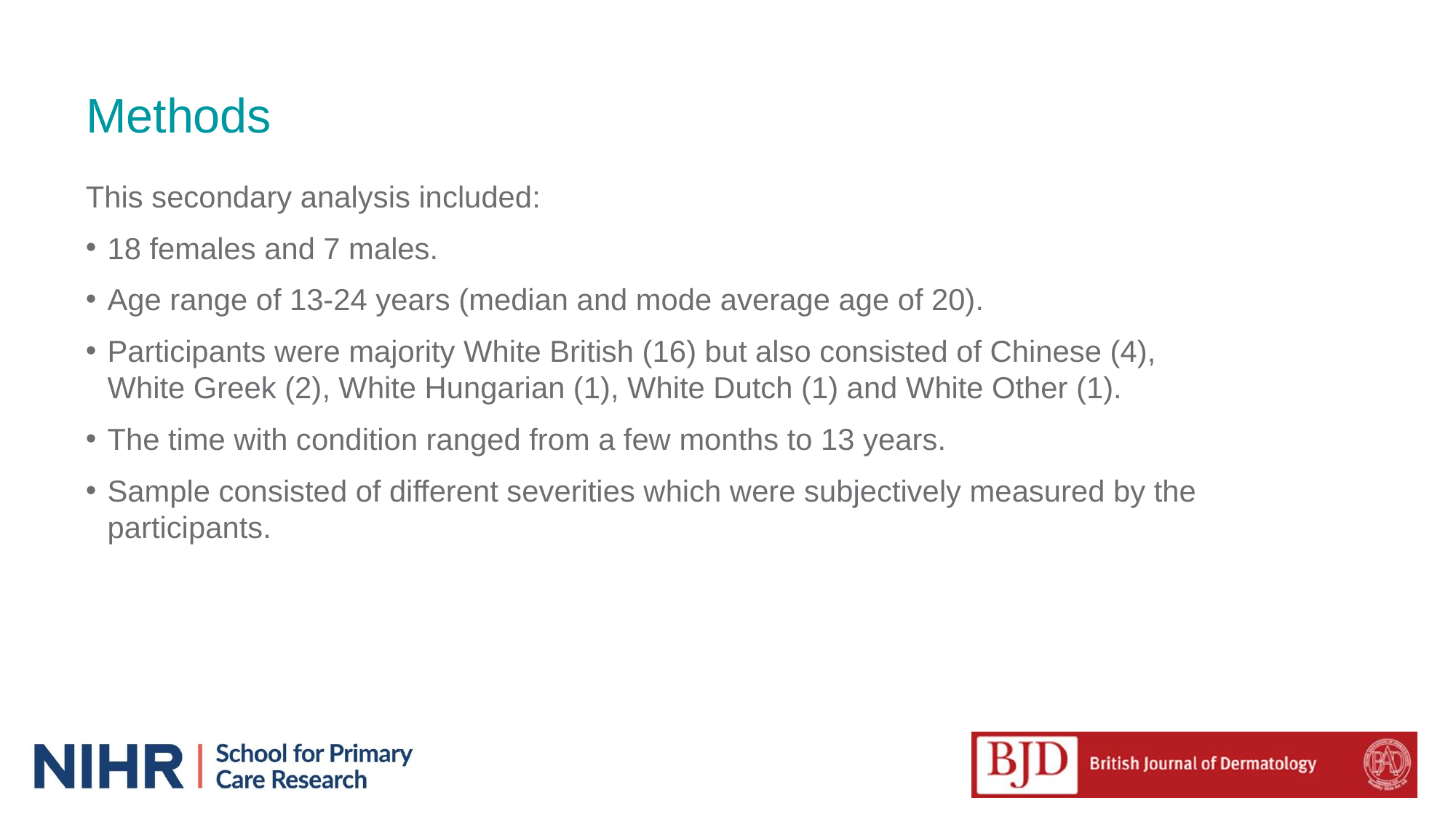

# Methods
This secondary analysis included:
18 females and 7 males.
Age range of 13-24 years (median and mode average age of 20).
Participants were majority White British (16) but also consisted of Chinese (4), White Greek (2), White Hungarian (1), White Dutch (1) and White Other (1).
The time with condition ranged from a few months to 13 years.
Sample consisted of different severities which were subjectively measured by the participants.

## Slide 6
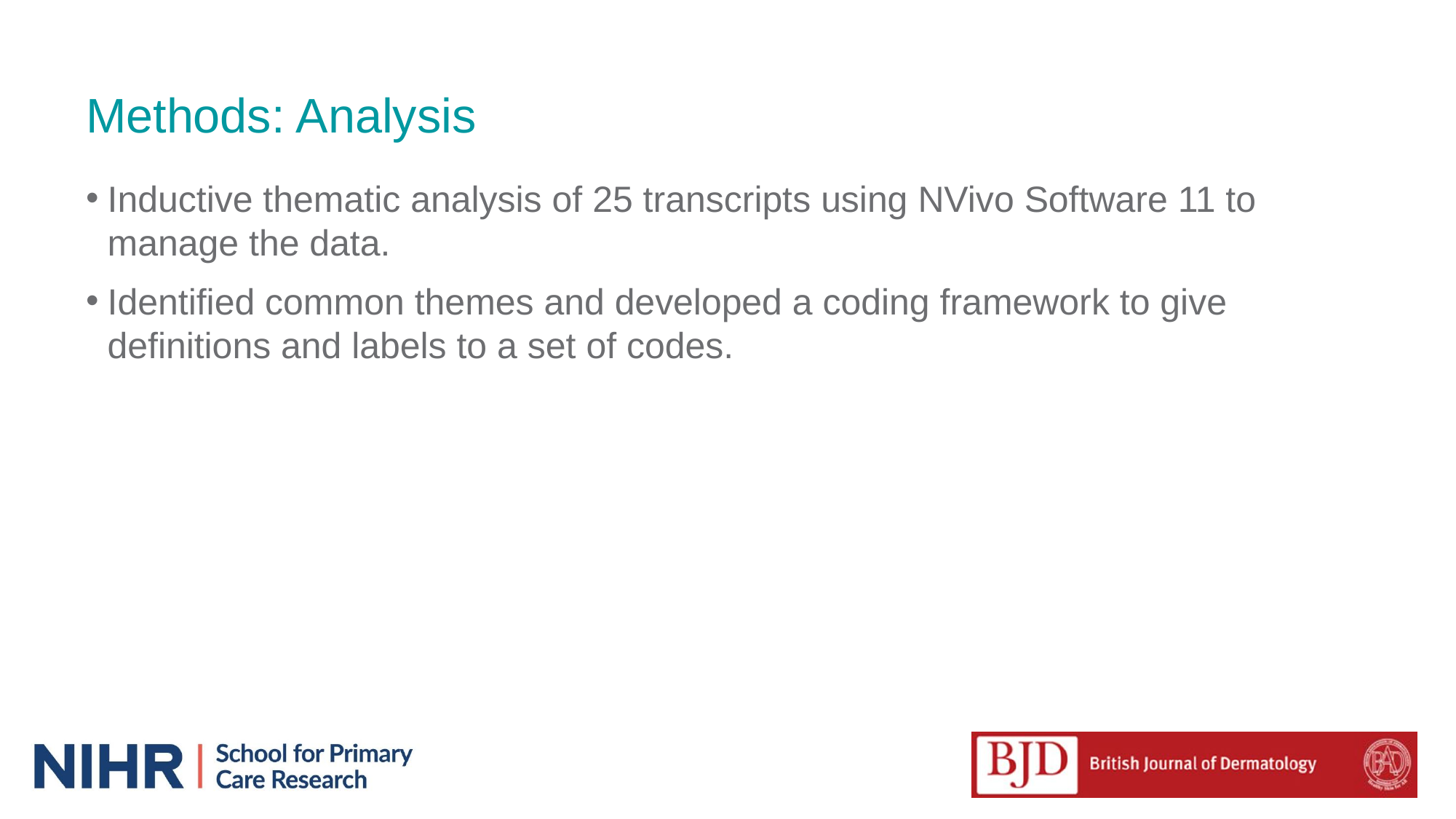

# Methods: Analysis
Inductive thematic analysis of 25 transcripts using NVivo Software 11 to manage the data.
Identified common themes and developed a coding framework to give definitions and labels to a set of codes.

## Slide 7
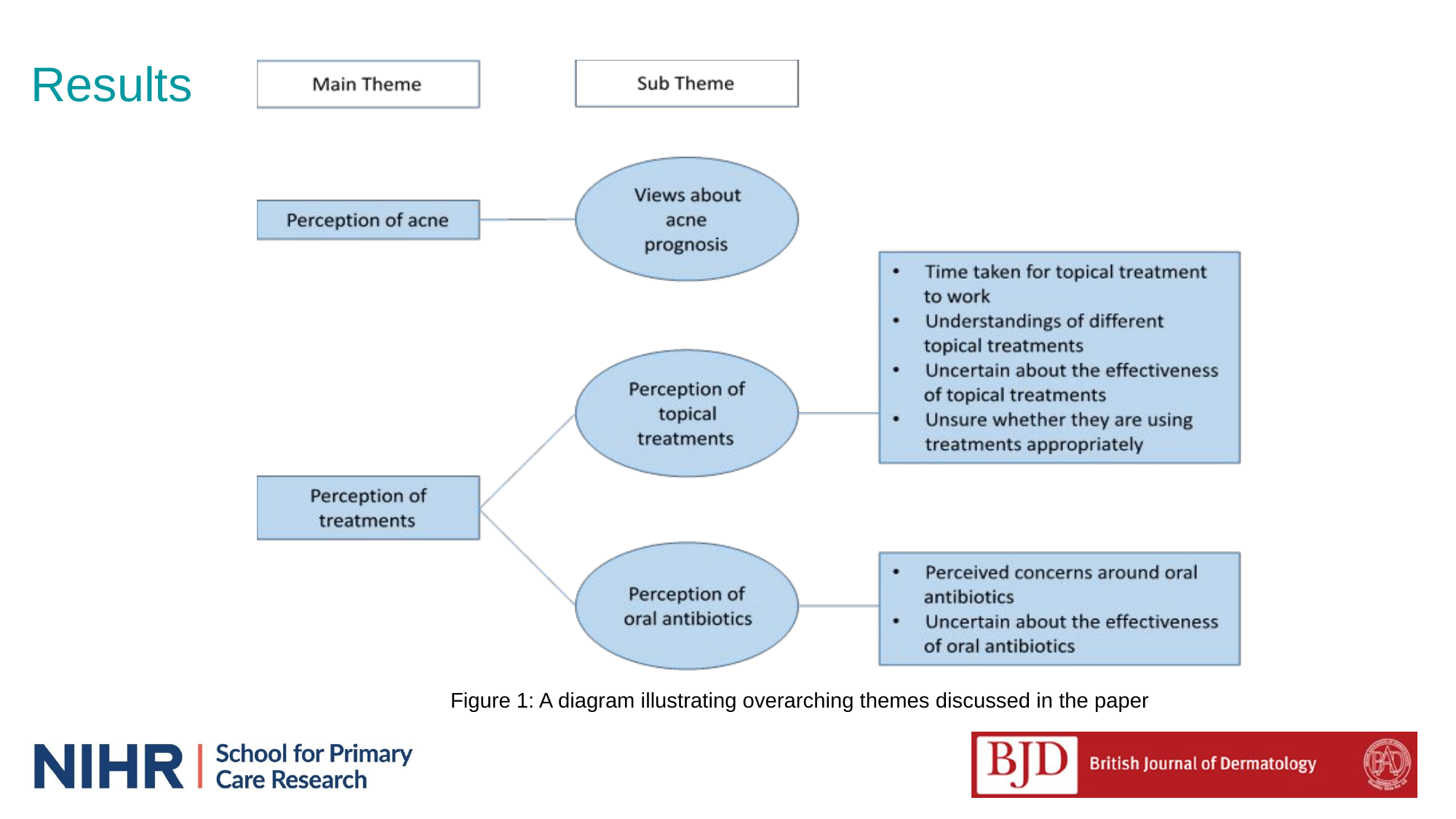

# Results
Figure 1: A diagram illustrating overarching themes discussed in the paper

## Slide 8
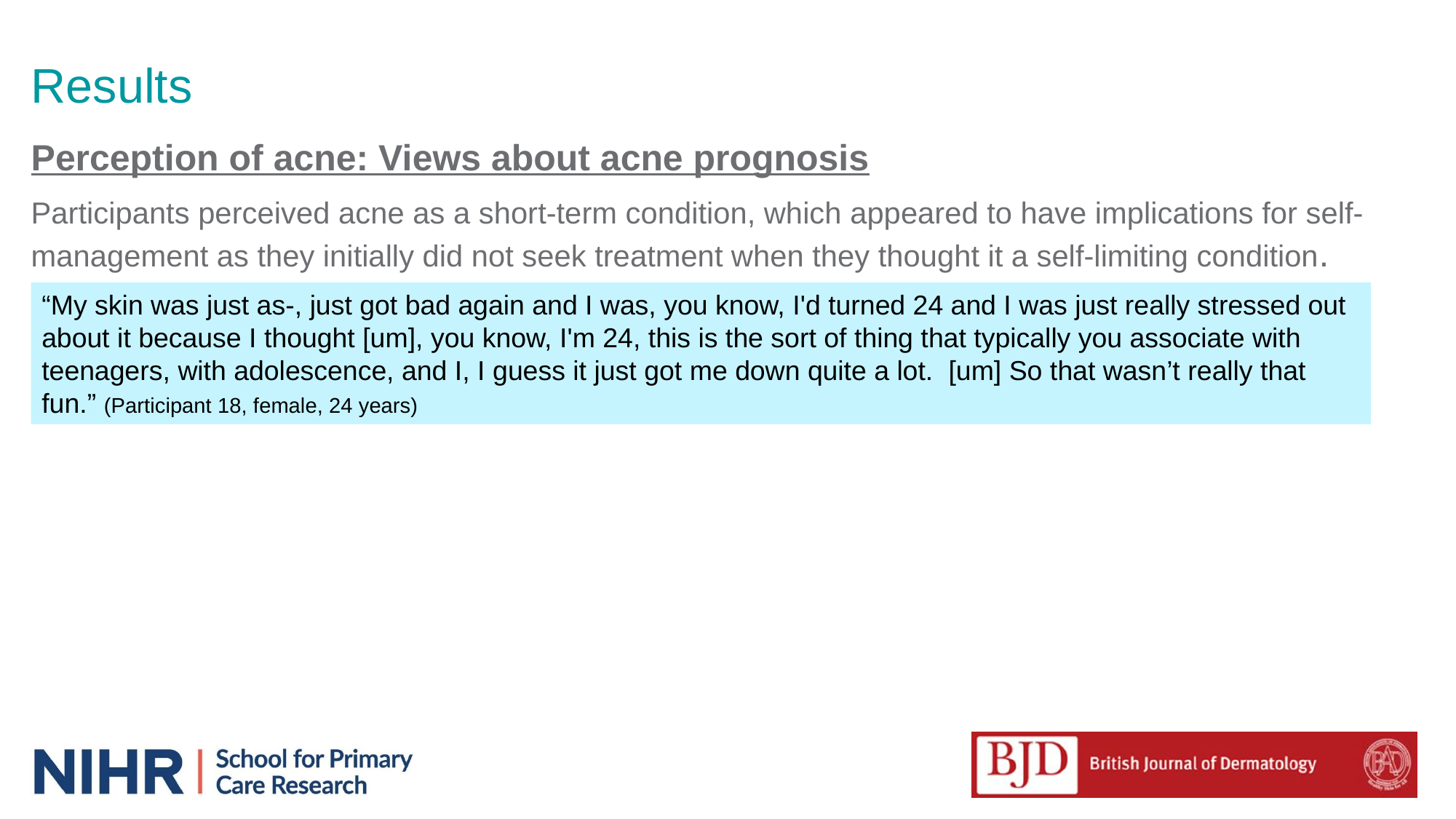

# Results
Perception of acne: Views about acne prognosis
Participants perceived acne as a short-term condition, which appeared to have implications for self-management as they initially did not seek treatment when they thought it a self-limiting condition.
“My skin was just as-, just got bad again and I was, you know, I'd turned 24 and I was just really stressed out about it because I thought [um], you know, I'm 24, this is the sort of thing that typically you associate with teenagers, with adolescence, and I, I guess it just got me down quite a lot. [um] So that wasn’t really that fun.” (Participant 18, female, 24 years)

## Slide 9
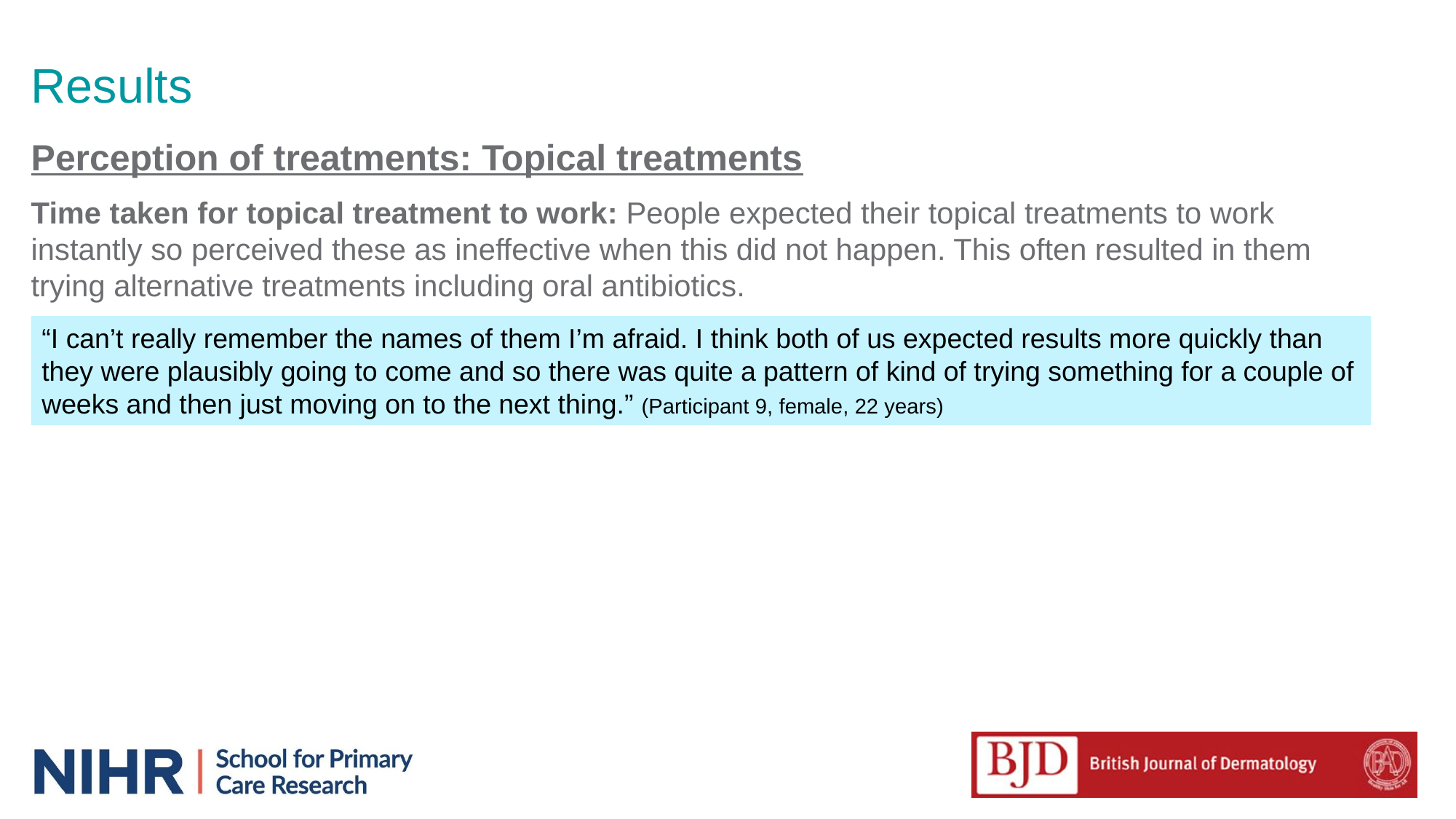

# Results
Perception of treatments: Topical treatments
Time taken for topical treatment to work: People expected their topical treatments to work instantly so perceived these as ineffective when this did not happen. This often resulted in them trying alternative treatments including oral antibiotics.
“I can’t really remember the names of them I’m afraid. I think both of us expected results more quickly than they were plausibly going to come and so there was quite a pattern of kind of trying something for a couple of weeks and then just moving on to the next thing.” (Participant 9, female, 22 years)

## Slide 10
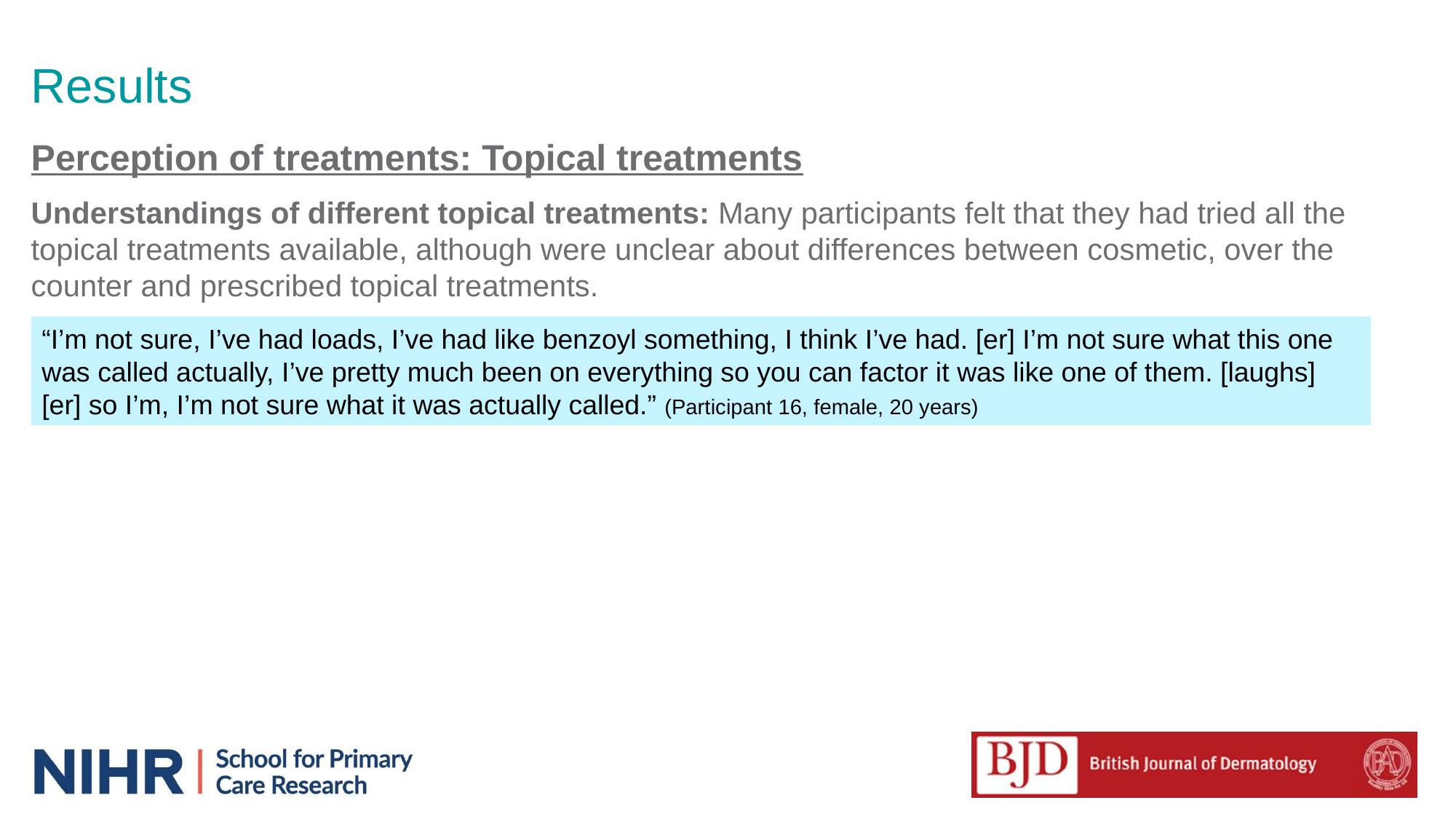

# Results
Perception of treatments: Topical treatments
Understandings of different topical treatments: Many participants felt that they had tried all the topical treatments available, although were unclear about differences between cosmetic, over the counter and prescribed topical treatments.
“I’m not sure, I’ve had loads, I’ve had like benzoyl something, I think I’ve had. [er] I’m not sure what this one was called actually, I’ve pretty much been on everything so you can factor it was like one of them. [laughs] [er] so I’m, I’m not sure what it was actually called.” (Participant 16, female, 20 years)

## Slide 11
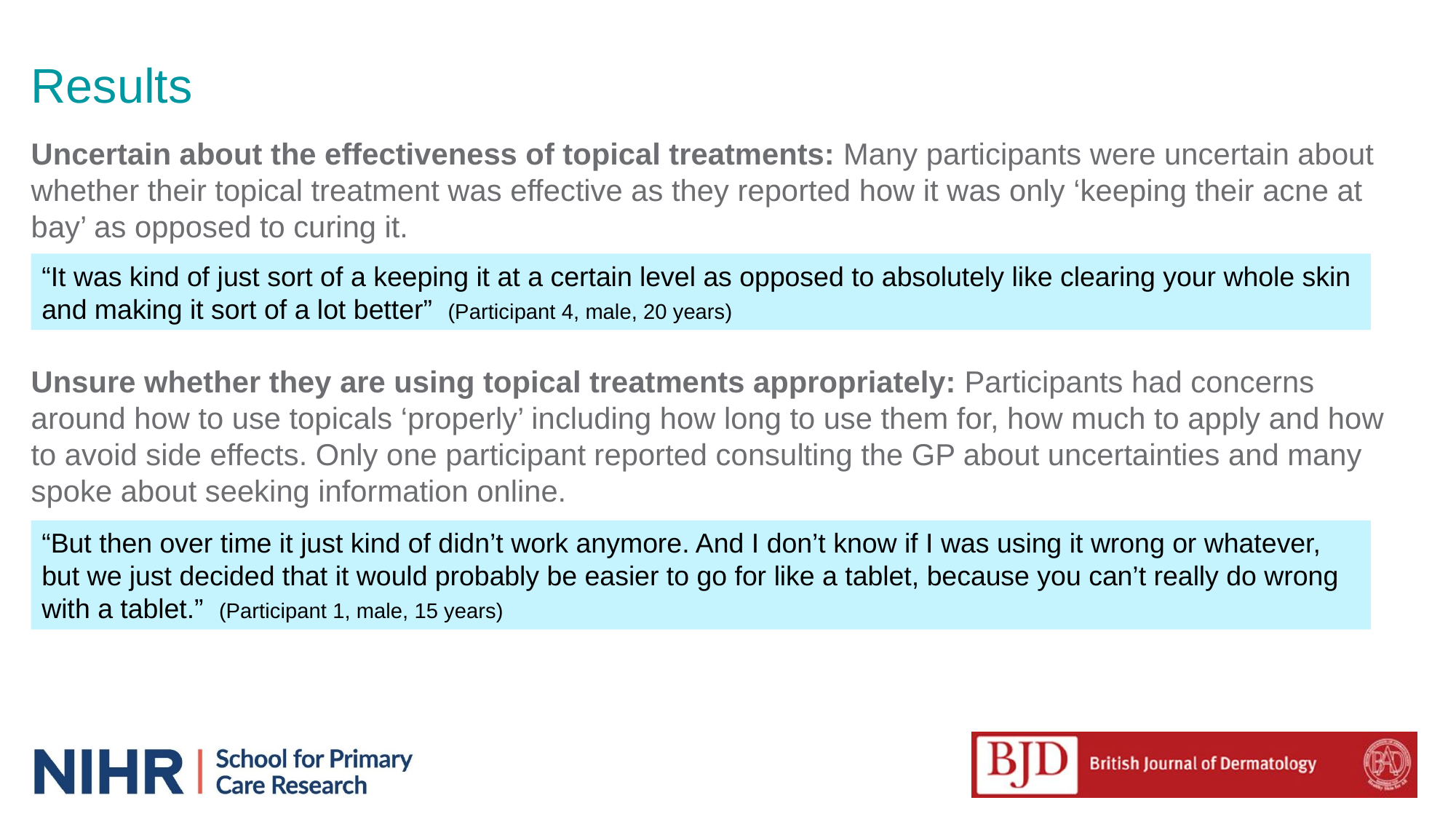

# Results
Uncertain about the effectiveness of topical treatments: Many participants were uncertain about whether their topical treatment was effective as they reported how it was only ‘keeping their acne at bay’ as opposed to curing it.
Unsure whether they are using topical treatments appropriately: Participants had concerns around how to use topicals ‘properly’ including how long to use them for, how much to apply and how to avoid side effects. Only one participant reported consulting the GP about uncertainties and many spoke about seeking information online.
“It was kind of just sort of a keeping it at a certain level as opposed to absolutely like clearing your whole skin and making it sort of a lot better” (Participant 4, male, 20 years)
“But then over time it just kind of didn’t work anymore. And I don’t know if I was using it wrong or whatever, but we just decided that it would probably be easier to go for like a tablet, because you can’t really do wrong with a tablet.” (Participant 1, male, 15 years)

## Slide 12
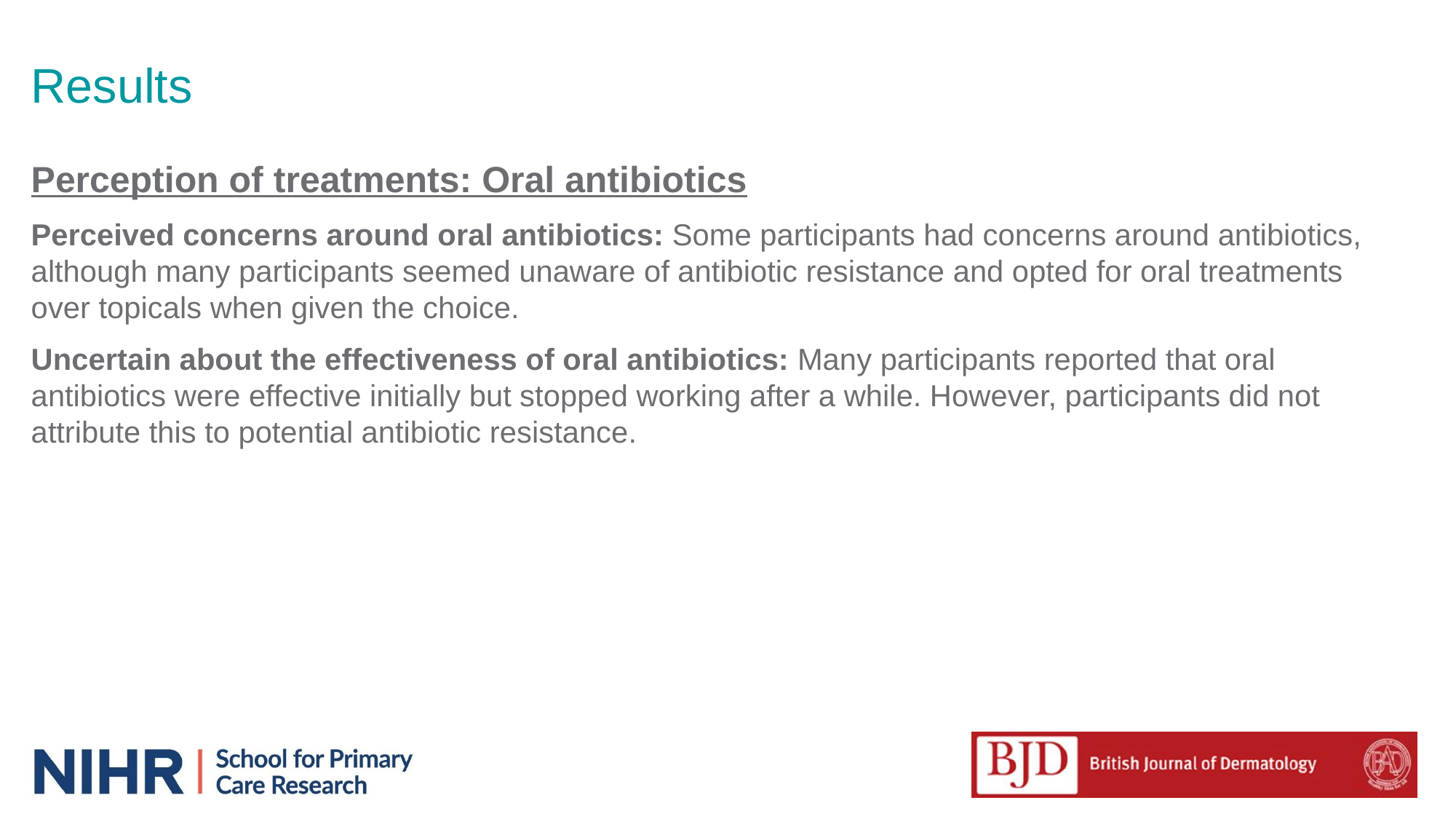

# Results
Perception of treatments: Oral antibiotics
Perceived concerns around oral antibiotics: Some participants had concerns around antibiotics, although many participants seemed unaware of antibiotic resistance and opted for oral treatments over topicals when given the choice.
Uncertain about the effectiveness of oral antibiotics: Many participants reported that oral antibiotics were effective initially but stopped working after a while. However, participants did not attribute this to potential antibiotic resistance.

## Slide 13
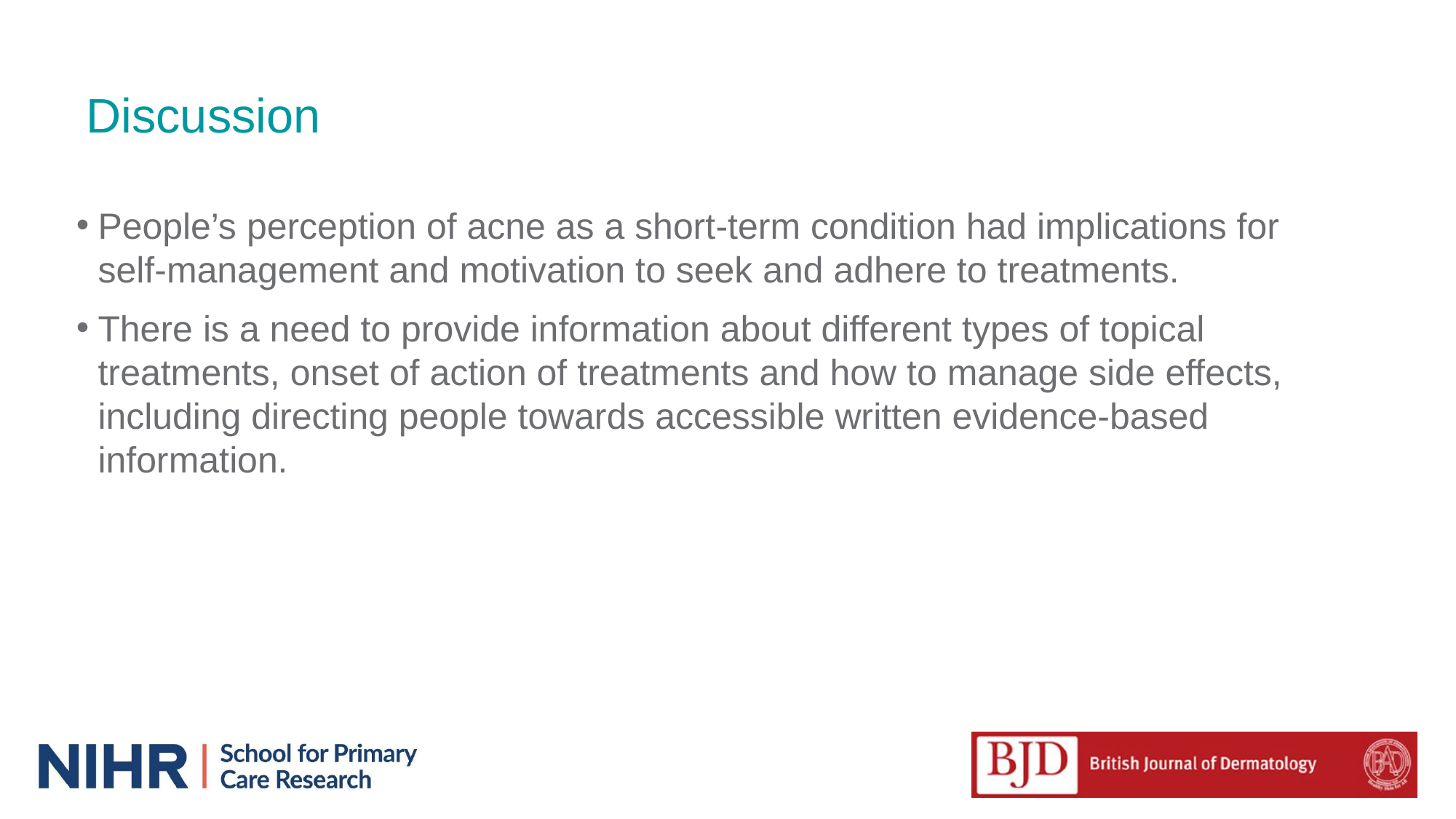

# Discussion
People’s perception of acne as a short-term condition had implications for self-management and motivation to seek and adhere to treatments.
There is a need to provide information about different types of topical treatments, onset of action of treatments and how to manage side effects, including directing people towards accessible written evidence-based information.

## Slide 14
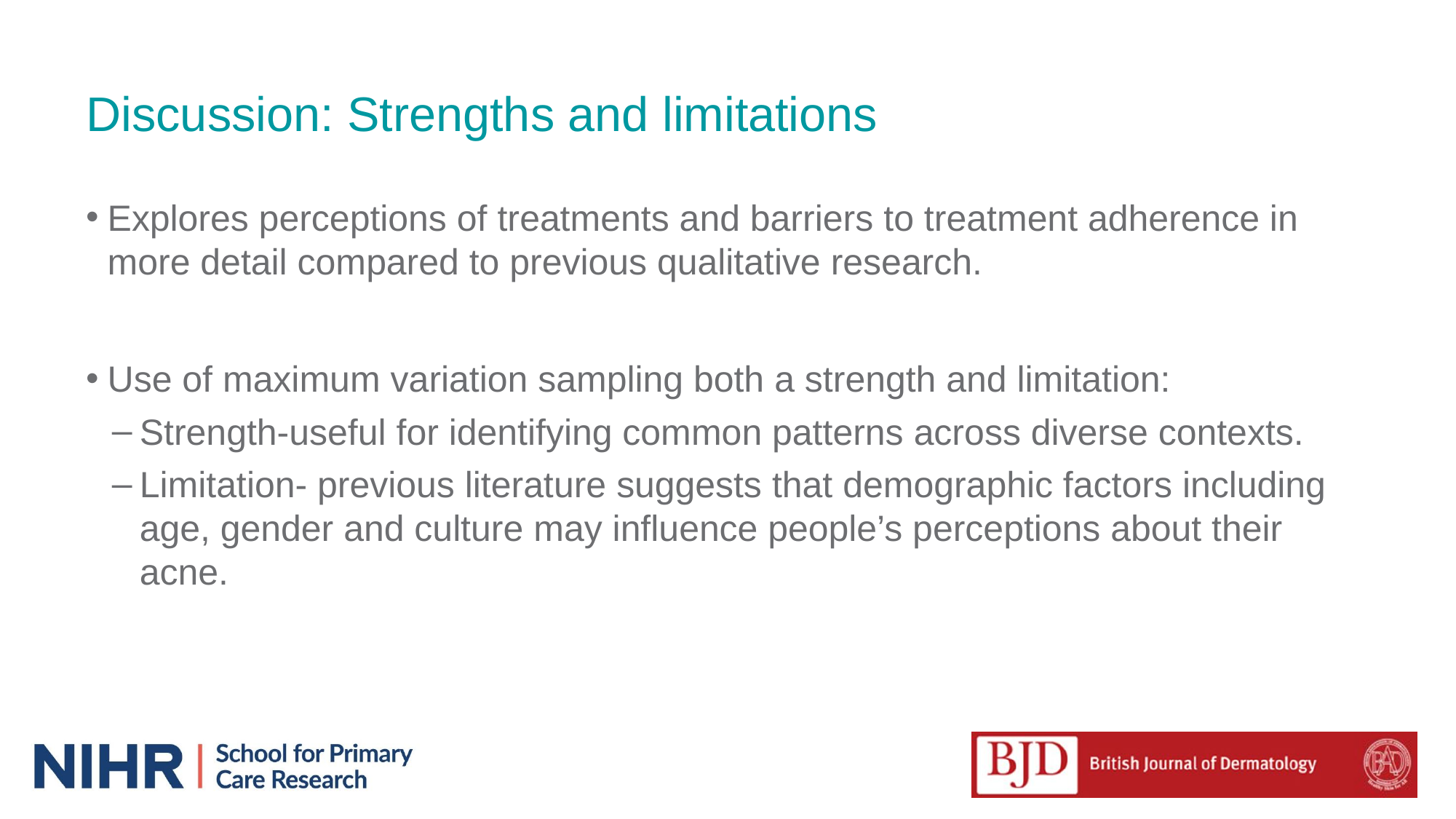

# Discussion: Strengths and limitations
Explores perceptions of treatments and barriers to treatment adherence in more detail compared to previous qualitative research.
Use of maximum variation sampling both a strength and limitation:
Strength-useful for identifying common patterns across diverse contexts.
Limitation- previous literature suggests that demographic factors including age, gender and culture may influence people’s perceptions about their acne.

## Slide 15
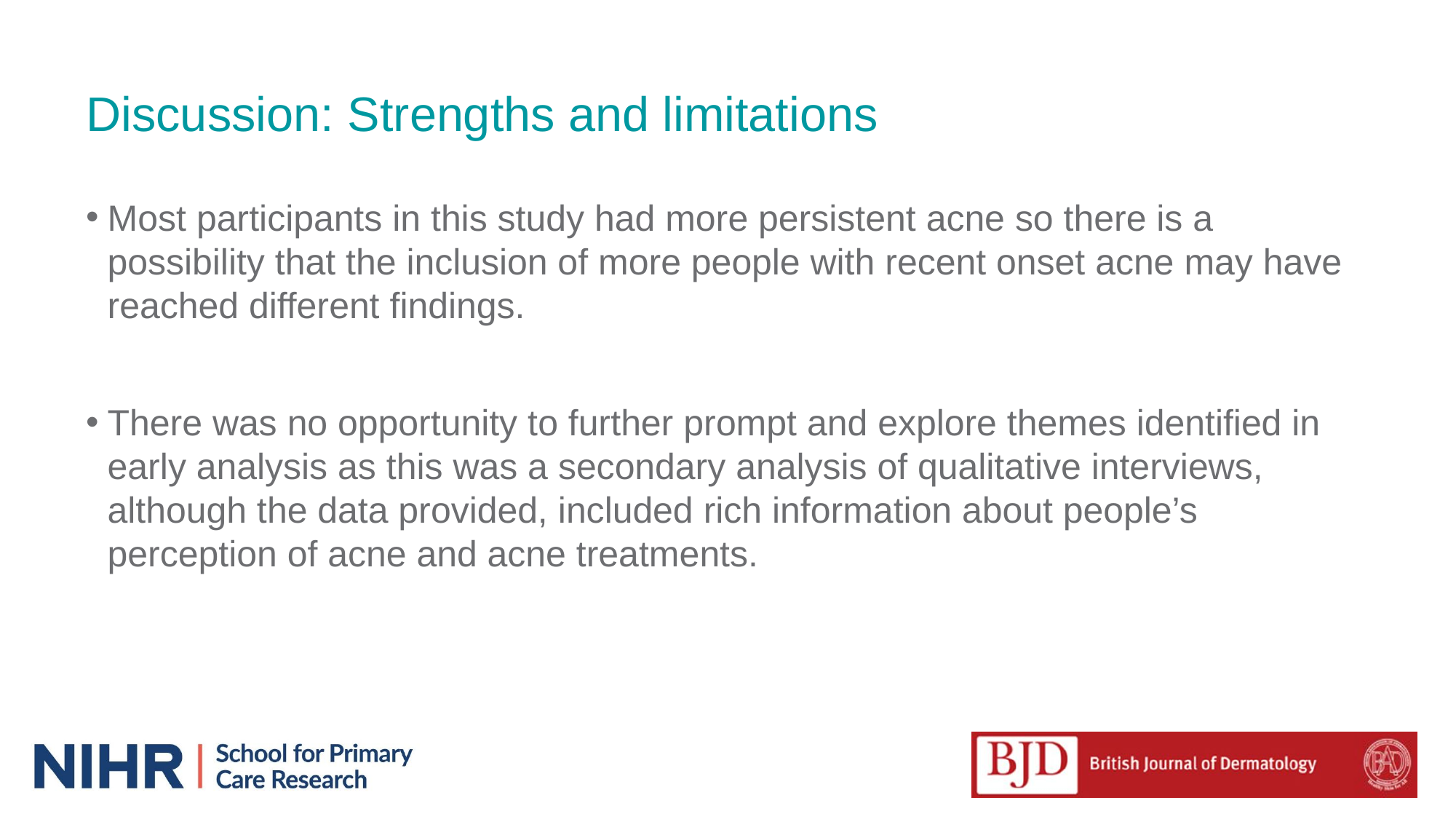

# Discussion: Strengths and limitations
Most participants in this study had more persistent acne so there is a possibility that the inclusion of more people with recent onset acne may have reached different findings.
There was no opportunity to further prompt and explore themes identified in early analysis as this was a secondary analysis of qualitative interviews, although the data provided, included rich information about people’s perception of acne and acne treatments.

## Slide 16
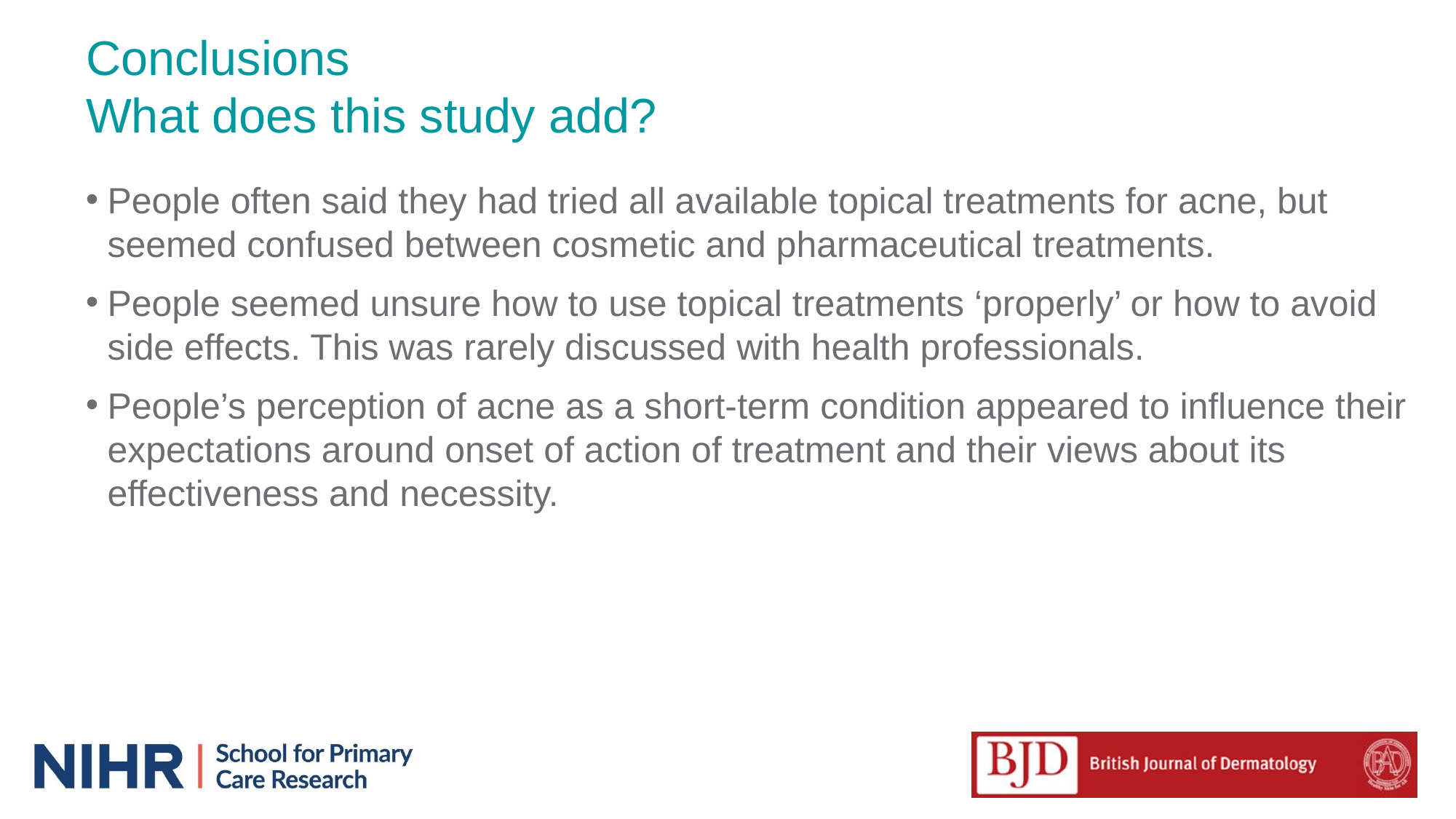

# Conclusions What does this study add?
People often said they had tried all available topical treatments for acne, but seemed confused between cosmetic and pharmaceutical treatments.
People seemed unsure how to use topical treatments ‘properly’ or how to avoid side effects. This was rarely discussed with health professionals.
People’s perception of acne as a short-term condition appeared to influence their expectations around onset of action of treatment and their views about its effectiveness and necessity.

## Slide 17
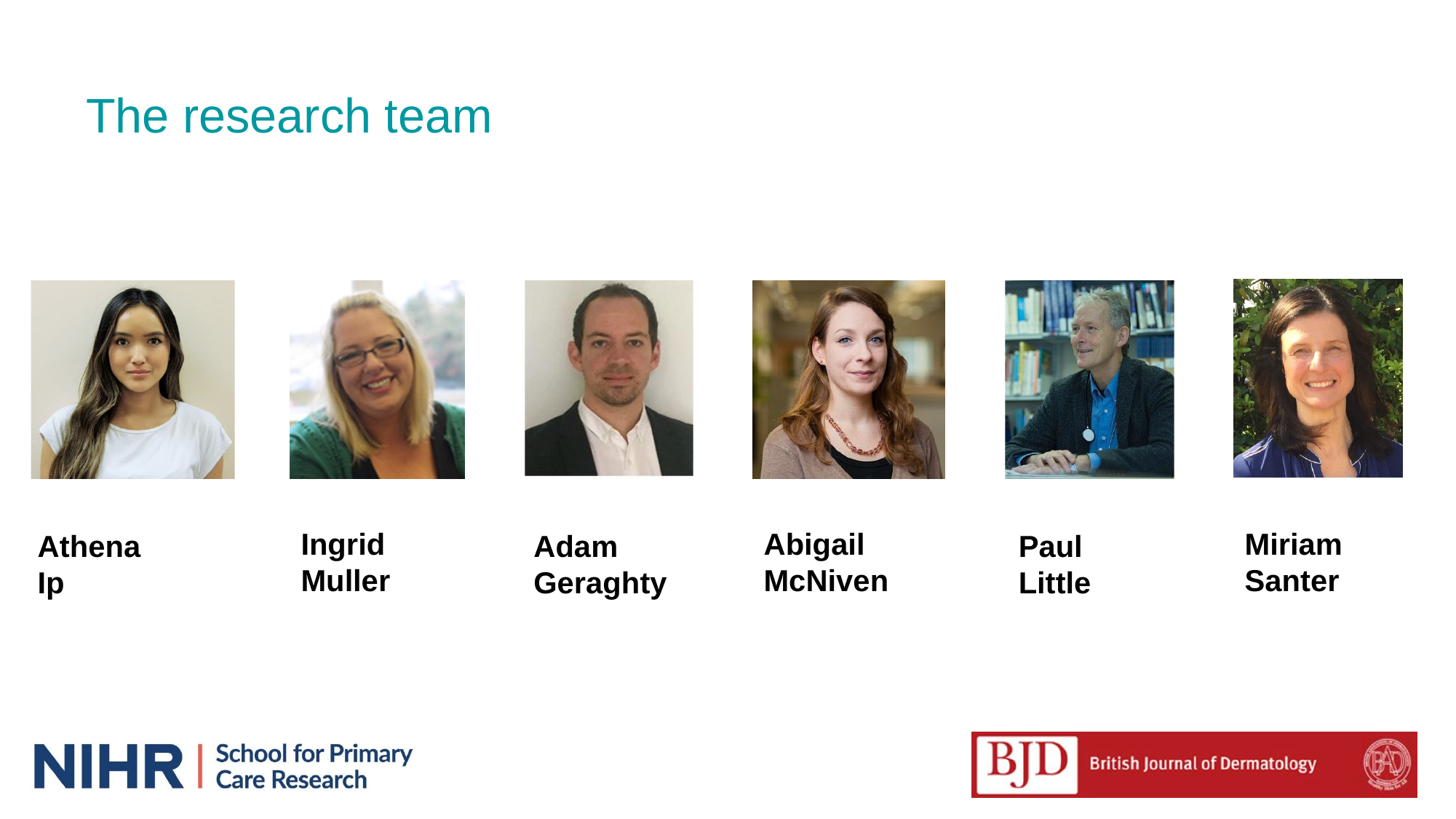

# The research team
Ingrid Muller
Abigail McNiven
Miriam Santer
Athena Ip
Adam Geraghty
Paul Little

## Slide 18
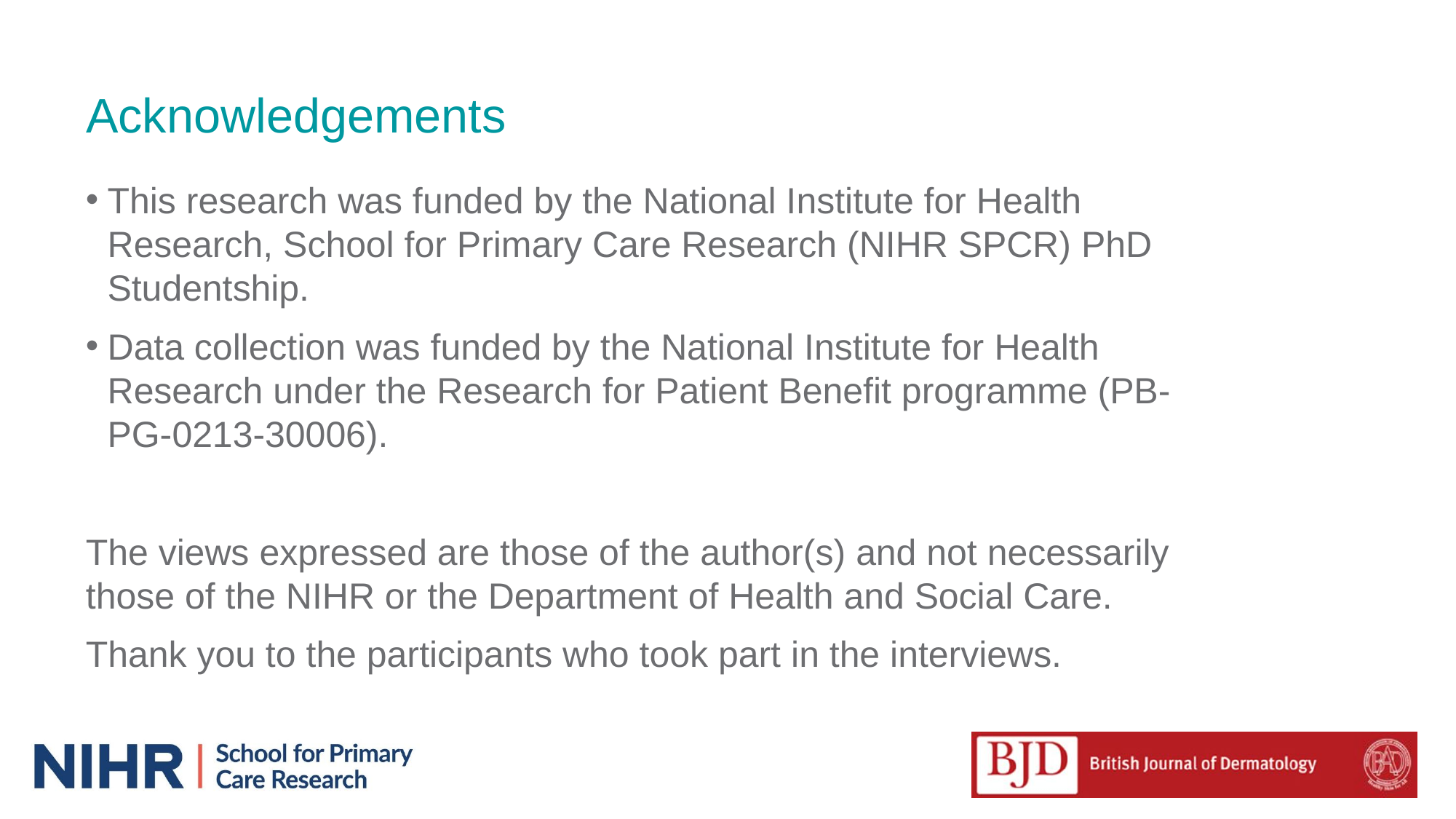

# Acknowledgements
This research was funded by the National Institute for Health Research, School for Primary Care Research (NIHR SPCR) PhD Studentship.
Data collection was funded by the National Institute for Health Research under the Research for Patient Benefit programme (PB-PG-0213-30006).
The views expressed are those of the author(s) and not necessarily those of the NIHR or the Department of Health and Social Care.
Thank you to the participants who took part in the interviews.

## Slide 19
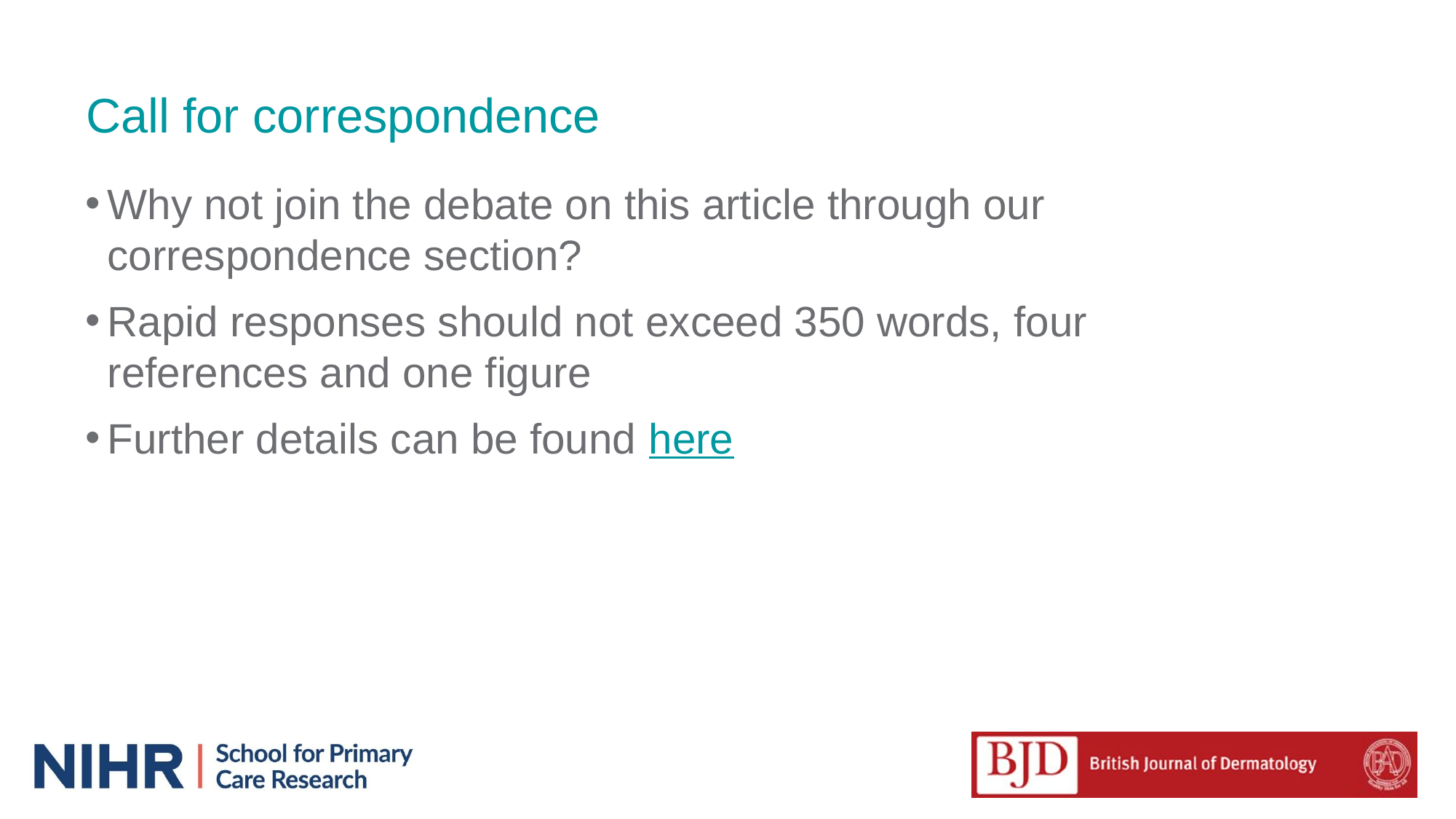

# Call for correspondence
Why not join the debate on this article through our correspondence section?
Rapid responses should not exceed 350 words, four references and one figure
Further details can be found here
